# The Endosymbiont *Hamiltonella* Increases the Growth Rate of Its Host *Bemisia tabaci* during Periods of Nutritional Stress

**DOI:** 10.1371/journal.pone.0089002

**Published:** 2014-02-18

**Authors:** Qi Su, Wen Xie, Shaoli Wang, Qingjun Wu, Baiming Liu, Yong Fang, Baoyun Xu, Youjun Zhang

**Affiliations:** 1 College of Plant Protection, Hunan Agricultural University, Changsha, Hunan, People’s Republic of China; 2 Department of Plant Protection, Institute of Vegetables and Flowers, Chinese Academy of Agricultural Sciences, Beijing, People’s Republic of China; University of Utah, United States of America

## Abstract

The whitefly *Bemisia tabaci* (Gennadius) (Hemiptera: Aleyrodidae) harbors several bacterial symbionts. Among the secondary (facultative) symbionts, *Hamiltonella* has high prevalence and high infection frequencies, suggesting that it may be important for the biology and ecology of its hosts. Previous reports indicated that *Hamiltonella* increases whitefly fitness and, based on the complete sequencing of its genome, may have the ability to synthesize cofactors and amino acids that are required by its host but that are not sufficiently synthesized by the host or by the primary endosymbiont, *Portiera*. Here, we assessed the effects of *Hamiltonella* infection on the growth of *B. tabaci* reared on low-, standard-, or high-nitrogen diets. When *B. tabaci* was reared on a standard-nitrogen diet, no cost or benefit was associated with *Hamiltonella* infection. But, if we reared whiteflies on low-nitrogen diets, *Hamiltonella*-infected whiteflies often grew better than uninfected whiteflies. Furthermore, nitrogen levels in field-collected whiteflies indicated that the nutritional conditions in the field were comparable to the low-nitrogen diet in our laboratory experiment. These data suggest that *Hamiltonella* may play a previously unrecognized role as a nutritional mutualist in *B. tabaci*.

## Introduction

Many insect species harbor intracellular, bacterial symbionts, and the interaction between bacterium and insect can be parasitic, mutualistic, or neutral [1]. These symbiotic bacteria, which are strictly maternally inherited, can affect insect defense against natural enemies and pathogens [2]–[6], dispersal behavior [7], pest status [8], thermal resistance [9], [10], virus vector competence [11], [12], reproduction (including sex ratio) [13], and body color [14].

By supplementing nutrients that are deficient in the diet of the insects, intracellular symbionts have contributed to the evolutionary success of their hosts [15], [16]. The most developed nutritional associations are found in the obligatory symbionts (which are also referred to as primary symbionts) that provide essential amino acids and certain vitamins to insects with specialized feeding behaviors such as plant-feeding aphids [17] and blood-feeding tsetse flies [18], [19]. Microbial symbionts, however, may also compensate for the nutrient deficiencies of less specialized herbivorous animals [16], [20]. For example, the intracellular endosymbionts *Blochmannia* in carpenter ants can provide essential amino acids that are deficient in the ant diet and that thereby sustain colony fitness [21]. Similarly, cockroaches harbor intracellular bacteria (*Blattabacterium* sp.), which apparently recycle uric acid reserves, providing the insect with usable nitrogenous compounds during times of nitrogen famine [22]–[24]. The latter study demonstrates that a symbiont’s contribution may only be apparent when the host is nutritionally compromised.

The current research concerns bacterial symbionts in the whitefly *Bemisia tabaci* (Gennadius) (Hemiptera: Aleyrodidae). *B. tabaci* is a cryptic species complex of major agricultural pests that affect a wide range of crop species [25], [26]. As phloem-feeders, whiteflies feed exclusively on plant sap, which is generally limited in nitrogen content [27], [28]. While plant sap easily fulfills the daily energetic needs by providing ample carbohydrates [29], plant sap is unlikely to satisfy the nitrogen requirements of reproductive *B. tabaci* females [15], [30]. Like many other sap-feeding insects, whiteflies have evolved ancient relationships with intracellular bacteria that reside within a brightly pigmented abdominal organ known as the bacteriome [15]. Such bacteria commonly provide essential nutrients by the synthesis of essential amino acids missing from the diet [15], [30]. Recent sequencing has revealed that the obligate symbiont of *B. tabaci*, “*Candidatus* Portiera aleyrodidarum”, has a very small genome that can synthesize certain essential amino acids as well as carotenoids [31], [32] but not other essential amino acids or cofactors [33]. The essential amino acids and cofactors not provided by *Portiera*, however, might be provided by other symbionts of *B. tabaci* including the facultative (secondary) endosymbiont, *Hamiltonella*. Recent results of genome sequencing indicate that *Hamiltonella* may be able to synthesize amino acids and cofactors [34]. Other recent research indicates that *Hamiltonella* increases whitefly fitness [35]. Together, the latter two studies are consistent with the hypothesis that *Hamiltonella* has nutritional roles in *B. tabaci*.

Although whitefly hosts are not dependent upon *Hamiltonella* for amino acid biosynthesis, the bacterium could supplement host stores of amino acids or play a role in nitrogen homeostasis. Here, we examine how *Hamiltonella* infection alters the growth of *B. tabaci* when the whitefly is supplied with low, standard, or high levels of nitrogen in its diet. Our goal is to test the hypothesis that *Hamiltonella* may function as a nutritional mutualist in whiteflies.

## Materials and Methods

### Ethics Statement

The research complies with all laws of the country (China) in which it was performed and was approved by the ‘Department of Scientific Management of Chinese Academy of Agricultural Sciences, China’ (permit number: 20090112). The authority responsible for a national park or other protected area of land or sea, the relevant regulatory body concerned with protection of wildlife. The four field populations in our study were farmlands and were all in the permitted range. The field studies did not involve endangered or protected species.

### Whitefly Colony

The laboratory population of *B. tabaci* Q (recently termed the Mediterranean species) used in this study was originally collected on poinsettia in Beijing, China, in 2009 and has been reared on poinsettia (*Euphorbia pulcherrima* Wild. ex Klotz.) at 26±2°C with a 12-h light/12-h dark photoperiod in individual insect-proof cages. Periodic diagnostic screening revealed that this population harbored only *Portiera* and *Hamiltonella* [12]. In our previous article, rifampicin treatments were performed to generate a genetically identical whitefly strain that dramatically reduced the *Hamiltonella* numbers, while the primary symbiont *Portiera* had an obligatory relationship with *B. tabaci* and could not be removed by antibiotic treatment [12]. With the possibility of the *Hamiltonella* recover, prior the experiment, the adults had received rifampicin-infused sucrose with 50 µg/ml of rifampicin for 48 h in three successive generations to persistently suppress the *Hamiltonella* number. In this way, *Portiera* was maintained in the strains of the whitefly *B. tabaci*, while *Hamiltonella* can be completely removed, and qPCR assays demonstrated that most of the adults in the F4 generation were *Hamiltonella*-free (Fig. S1).

Four *B. tabaci* field populations from Haidian, Changping, Langfang, and Nankou, respectively were collected near Beijing, China during the 2013 crop season. At each site, three subsamples of whiteflies were collected, with an approximately 500–1000 m distance between each subsample. The whiteflies from three subsamples were combined into one collection per site. At least 100 whiteflies per collection site were preserved in 95% ethanol and stored at −20°C.

### Biotype and Symbiont Determination for *B. tabaci* Field Collection

Genomic DNA extraction from each of 30 individual whiteflies per collection site was performed on individual adult whiteflies as described by White et al. (2009) [37]. The whitefly biotype was determined by the CAPS (cleavage amplified polymorphic sequence) of *mtCOI* (mitochondrial cytochrome oxidase I) with the restriction endonucleases *Vsp*I [38]. The presence of *Hamiltonella* was determined using a diagnostic PCR protocol according to Pan et al. (2012) [39]. Reactions were performed in 25 µL volume containing 2.5 µL 10×PCR Buffer (Mg^2+^ Plus), 2 µL dNTP mix (2.5 mM of each nucleotide), 0.5 µL of each primer (10 µM each), and 0.125 µL of TaKaRa Taq (5 U/µL) (TaKaRa Biotechnology (Dalian) Co., Ltd). The PCR cycling conditions for detection of these symbionts were summarized in Pan et al. (2012) [39]. All PCRs included negative and positive controls. The resultant PCR products were electrophoresed on a 2.0% agarose gel in a 0.5×TBE buffer and visualized by Gelview staining. Because the obligate symbiont *Portiera* should be present in all extractions, any samples that failed to amplify *Portiera* were considered to be of poor quality and discarded.

### Whitefly Growth Rate Assays

The effect of *Hamiltonella* infection and nitrogen supply on the growth rate of *B. tabaci* Q was assessed using whiteflies that were three generations removed from the last rifampicin treatment. Newly-emerged adult females of *B. tabaci* were reared on four types of artificial diets. The standard diet contained 30% (w/v) sucrose and 5% yeast extract (YE) solution (Oxoid, Hampshire, England) in distilled water; this diet supports maximum survival of whiteflies [36]. A low-nitrogen diet contained 2% YE and 30% sucrose, and a high-nitrogen diet contained 10% YE and 30% sucrose. Finally, a no-nitrogen diet contained only 30% sucrose. Stock solutions of the diets were prepared under aseptic conditions using double-distilled water (DDW) and were sterilized by autoclaving.

Female whiteflies (genetically identical and differing only in the presence or absence of *Hamiltonella* infection) were reared individually on the four diets described in the previous section for 5 days after adult eclosion. The diets were provided in Parafilm-membrane sachets in a feeding chamber [12]. An MT5 microbalance (Mettler) was used to weigh each whitefly to the nearest microgram at the start and at the end of the 5-day feeding period. Whitefly relative growth rate (RGR) was assessed by the formula ln (weight on day 5/weight on day 0)/5. A separate experiment was performed for the high-, low-, and no-nitrogen diet, and in each case the standard diet was included as a control. These experiments were performed three times (trials 1, 2, and 3) with the high-nitrogen, low-nitrogen and no-nitrogen diets, respectively.

### Amino Acid Analysis

Laboratory *Hamiltonella*-infected female whiteflies that were reared on the three types of diets (high, standard, and low nitrogen) or that were collected from the four field sites were subjected to amino acid analysis in batches (50 adult females per batch, six replicate pools per combination of *Hamiltonella* infection status and diet or per field site). Each batch was homogenized in 0.1 ml of ice-cold 80% ethanol in a glass, hand-held tissue grinder. After centrifugation at 12,000 g for 15 min to remove debris and precipitated protein, the supernatant was retained for subsequent amino acid analysis. The 800 µL aliquots of extract were dried and hydrolysed in 6 mol l^−1^ HCl at 110°C for 24 h in a sealed ampoule. The hydrolysate was neutralised with NaOH, dried in a Speed-Vac and dissolved in 80% ethanol, and then filtrated through 0.45 µm membrane. The total content of free amino acids in each supernatant was determined with a Sykam S-433 D automatic amino acid analyzer (Sykam, Eresing, Germany).

### Statistical Analysis

In the three laboratory experiments concerning the effect of *Hamiltonella* and diet on growth rate, growth rates between *Hamiltonella*-infected and noninfected *B. tabaci* Q were compared with independent sample *t-*tests. This was done separately for each trial and was also done to compare growth rates of infected and noninfected whiteflies on the standard diet (the control) that was included in each trial. Data for amino acid content were subjected to a one-way ANOVA; if the ANOVA was significant, means were compared with a Tukey’s HSD test. All data were analyzed using SPSS software package (ver.17, SPSS Inc, USA). Statistical significance was determined at *P*<0.05. Means and standard errors are reported.

## Results

### Survey of Biotype and Symbionts for *B. tabaci* Field Collection

All the 4 field populations were of pure Q biotype and all individuals of the 4 field populations had *Portiera* and 87% individuals had *Hamiltonella* (Data not shown).

### Whitefly Growth Rate as Affected by *Hamiltonella* Infection and Nitrogen Level in an Artificial Diet

**Figure 1:**
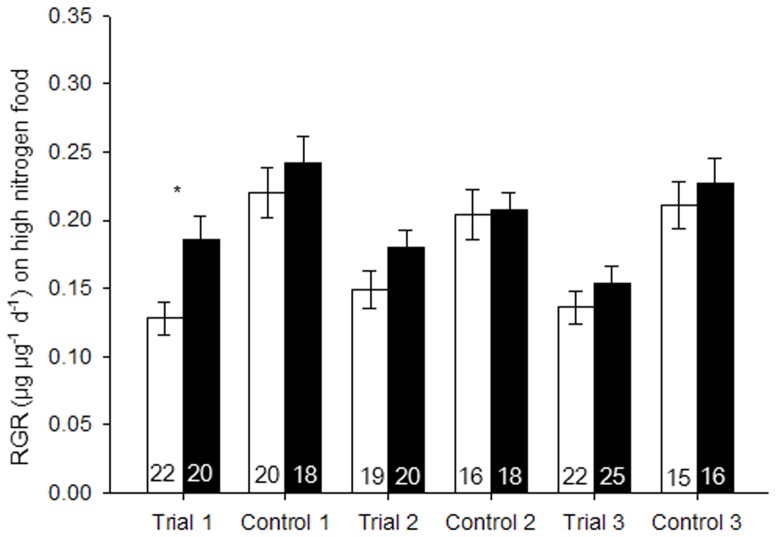
Mean relative growth rate (RGR) of *B. tabaci* females reared on standard diet (control) or high-nitrogen diet. Black bars and white bars indicate infected and noninfected females, respectively. Replicate numbers are noted within the columns. Values are means±SE. The experiment was performed three times (trial 1–3). For each paired comparison (± infection), asterisks indicate a significant difference (*, *p*<0.05) based on a *t*-test.

**Figure 2:**
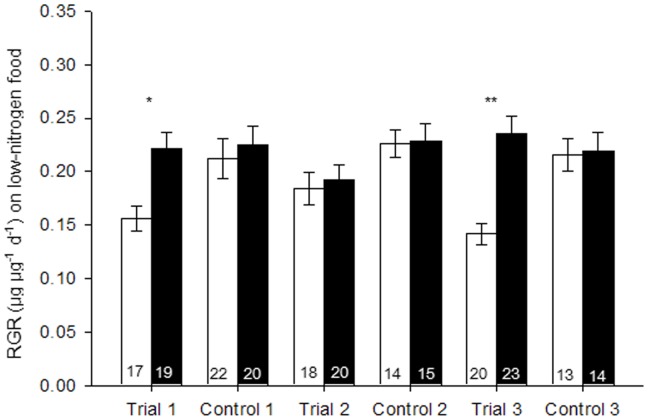
Mean relative growth rate (RGR) of *B. tabaci* females reared on standard diet (control) or low-nitrogen diet. Black bars and white bars indicate infected and noninfected females, respectively. Replicate numbers are noted within the columns. Values are means±SE. The experiment was performed three times (trial 1–3). For each paired comparison (± infection), asterisks indicate a significant difference (*, *p*<0.05; **, *p*<0.01) based on a *t*-test.

**Figure 3:**
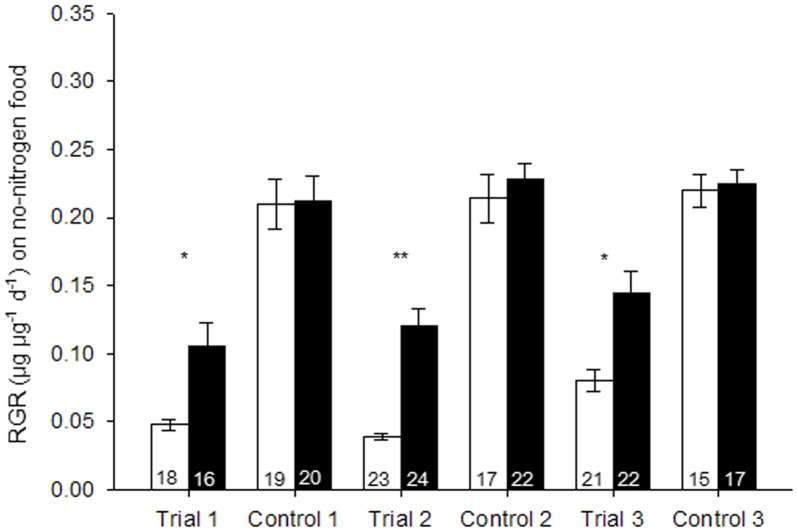
Mean relative growth rate (RGR) of *B. tabaci* females reared on standard diet (control) or no-nitrogen diet. Black bars and white bars indicate infected and noninfected females, respectively. Replicate numbers are noted within the columns. Values are means±SE. The experiment was performed three times (trial 1–3). For each paired comparison (± infection), asterisks indicate a significant difference (*, *p*<0.05; **, *p*<0.01) based on a *t*-test.

When *B. tabaci* females were reared on the standard diet (control), the RGR showed no significant difference between *Hamiltonella*-infected and noninfected whiteflies in any trials of the three experiments (Fig. 1–3). When *B. tabaci* females were reared on diets that contained high levels of nitrogen, *Hamiltonella*-infected whiteflies showed 45% higher RGR than did noninfected whiteflies in trial 1, but it showed no differences in trial 2 and 3 (Fig. 1). On a low-nitrogen diet, the RGR of *Hamiltonella*-infected whiteflies was 42% and 65% higher than that of noninfected ones in trial 1 and 3, respectively, but showed no difference in trial 2 (Fig. 2). On a no-nitrogen diet, the RGR of *Hamiltonella*-infected whiteflies were significant higher than that of noninfected ones in all three trials (Fig. 3). Regardless of diet, RGR was never lower for *Hamiltonella*-infected than noninfected *B. tabaci* females (Fig. 1–3).

### Total Amino Acid Content in Adult *B. tabaci* Females Reared in the Laboratory or Collected from the Field

**Figure 4:**
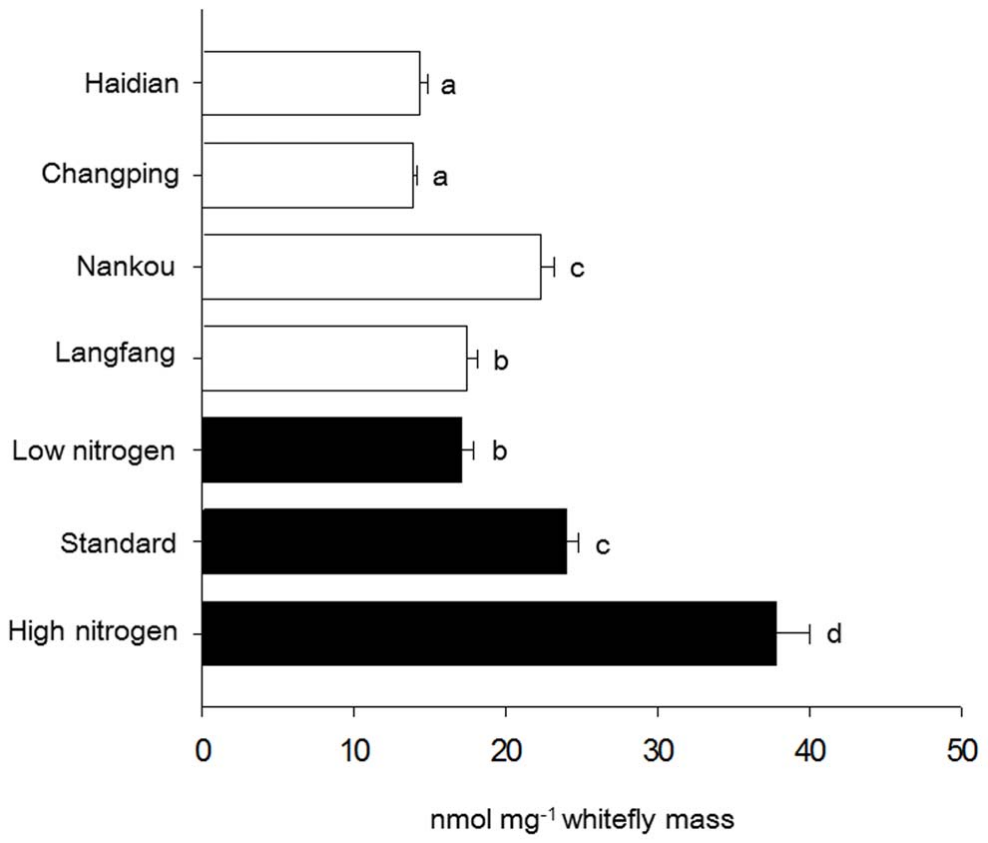
Total amino acid content of adult *B. tabaci* females. Field collected whiteflies are represented by white bars and lab reared whiteflies by black bars. Values are means+SE. Means followed by the same letter are not significantly different at *p*<0.05 according to ANOVA.

The free amino acid content in the *Hamiltonella*-infected whiteflies reared in laboratory was positively correlated with the total nitrogen concentration in the diet (Fig. 4). In addition, *Hamiltonella* did not significantly influence the free amino acid content of the whiteflies reared on standard diets (*t*-test, *p*>0.05); the free amino acid content was 21.14±1.30 nmol mg^−1^ whitefly mass with *Hamiltonella* infection and 25.47±1.60 nmol mg^−1^ whitefly mass without *Hamiltonella* infection.

The free amino acid content of adult female whiteflies collected from three of the four field sites (Haidian, Changping, and Langfang) was less than or similar to that for whiteflies reared on the low-nitrogen diet in the laboratory (Fig. 4). The free amino acid content was higher in whiteflies at Nankou than at the other three field sites and was similar to that observed for whiteflies reared on the standard diet.

## Discussion

Metabolic provisioning of hosts by endosymbionts is common in obligate associations [17], [40]. Analysis of the genome sequence of the obligate symbiont of *B. tabaci* revealed that it lacks metabolic pathways for cofactors and some essential amino acids [33]. These results suggested that the genome of the insect or of the secondary endosymbionts might encode the enzymes needed to synthesize the missing cofactors and amino acids. As shown in another report, these missing metabolic pathways might exist in *Hamiltonella* [34]. Given the predictions of nitrogen as potential interaction points for *Hamiltonella* and their hosts, we experimentally determined whether *Hamiltonella* could influence the nitrogen homeostasis and growth of *B. tabaci* females. Our results demonstrate that *Hamiltonella* which naturally infects *B. tabaci* can act as a nutritional mutualist, i.e., *Hamiltonella* significantly increased the growth of *B. tabaci* when the whiteflies were subjected to low nitrogen environments. The results obtained with the low-nitrogen diet seem most relevant because field-collected whiteflies contained low amounts of amino acids. Although decreases in dietary nitrogen severely reduced the growth of both infected and noninfected females in our laboratory experiments, the reduction was less for infected females than for noninfected females, suggesting that *Hamiltonella* might provide protection against nitrogen deficiency. This is the first report of *Hamiltonella* having a compensatory effect on an insect host during periods of nutritional deficiency.

Another recent study from our laboratory demonstrated that *Hamiltonella* infection could substantially enhance whitefly performance; when the bacterium was removed by antibiotic treatment, adult development was impaired and reproductive output was reduced [35]. Considering the latter results and those provided in the current report, it seems clear that *Hamiltonella* infection increases host fitness. Increases in *B. tabaci* fitness also occur with infection by *Rickettsia*, which is another vertically transmitted endosymbiotic bacterium [13]. While insects may often benefit from harboring symbionts, they also may experience a cost [41]–[44]. The cost can be greater in older animals [45], and in contrast to the results reported here, the costs can sometimes be greater in nutritionally stressed animals [43]. Whereas most of the time they may gain the main benefits from their bacterial companions (e.g. nutrient provisioning and nitrogen recycling) [46]–[49].

The supplement or complement the essential nutrients provided by a primary symbiont was also reported for secondary symbiont in sharpshooters [50], cedar aphid [51], and pea aphid [52]. In the case of two endosymbiotic bacteria in the sharp shooter *Homalodisca vitripennis*, McCutcheon and Moran (2007) [53] showed that coevolution resulted in complementarity of function in that one bacterium encodes for metabolic pathways that are missing in the other and in the insect. Given the results of the current study and given that *Hamiltonella* inhabits bacteriocytes alongside *Portiera* [35], [54], [55], we suspect that the primary endosymbiont *Portiera* and the second endosymbiont *Hamiltonella* may perform complementary functions in *B*. *tabaci* hosts.

Taken together, our findings suggest that *Hamiltonella* may produce essential nutrients that are not produced or are insufficiently produced by *B. tabaci* or *Portiera* under nutrient stress. If this inference is correct, it could explain the high frequency of *Hamiltonella* in whitefly populations in China [39], [56] and would also suggest that *Hamiltonella* might be considered an obligate or nearly obligate endosymbiont from the perspective of the whitefly. *Hamiltonella* spp. is the main bacterium associated with whiteflies [57]–[60] but does not obligatorily depend on the whitefly hosts for survival [30]. Within the whitefly host, however, this bacterium enjoys a secure and stable environment with an abundance of nutrients and is also provided with a ready means of dispersal. That this bacterium is present in all stages of the *B. tabaci* life cycle (QS, unpublished data), that at least some elements are transmitted effectively from parents to offspring, and that this bacterium increases host fitness in general and increases the host growth rate under nutrient stress suggests that the association benefits both partners.

## Supporting Information

Figure S1 ***Portiera*** **and** ***Hamiltonella*** **densities across treatments with antibiotics.** To quantify *Portiera* and *Hamiltonella*, total DNA was extracted and used for quantitative PCR. The mean number of genome of *Portiera* and *Hamiltonella* was given per *actin* copies.
(TIF)
