# Supplementary figures and images for: The Endosymbiont Hamiltonella Increases the Growth Rate of Its Host Bemisia tabaci during Periods of Nutritional Stress
Source: PLoS One. 2014 Feb 18;9(2):e89002. doi: 10.1371/journal.pone.0089002 (PMC3928334; doi:10.1371/journal.pone.0089002)

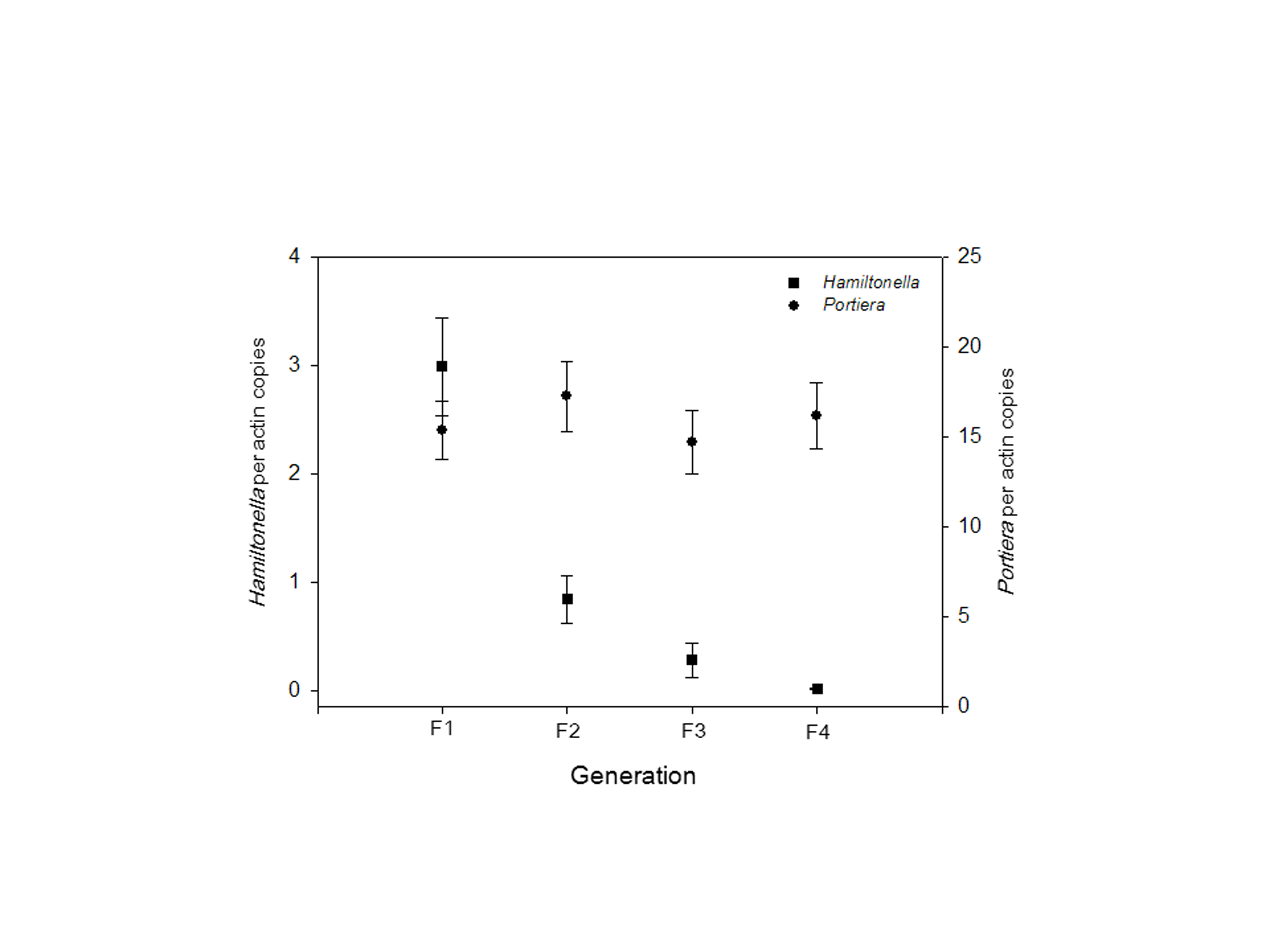

Supplement: Figure S1 — Portiera and Hamiltonella densities across treatments with antibiotics. To quantify Portiera and Hamiltonella, total DNA was extracted and used for quantitative PCR. The mean number of genome of Portiera and Hamiltonella was given per actin copies. (TIF) [file pone.0089002.s001.tif]
